# Joint effects of recent stressful life events and adverse childhood experiences on perinatal comorbid anxiety and depression

**DOI:** 10.1186/s12884-023-05375-1

**Published:** 2023-01-18

**Authors:** Yanyan Hou, Mengqing Shang, Xiayan Yu, Yue Gu, Haiyan Li, Mengjuan Lu, Minmin Jiang, Hualong Zhen, Beibei Zhu, Fangbiao Tao

**Affiliations:** 1 Department of Maternal, Child and Adolescent Health, School of Public Health, Anhui Medical University, No 81 Meishan Road, 230032 Hefei, Anhui China; 2 Key Laboratory of Population Health Across Life Cycle (Anhui Medical University), Ministry of Education of the People’s Republic of China, No 81 Meishan Road, 230032 Hefei, Anhui China; 3 Anhui Provincial Key Laboratory of Population Health and Aristogenics, Anhui Medical University, No 81 Meishan Road, 230032 Hefei, Anhui China; 4 NHC Key Laboratory of Study on Abnormal Gametes and Reproductive Tract, No 81 Meishan Road, 230032 Hefei, Anhui China; 5 Department of Occupational Health and Environmental Health, School of Public Health, Anhui Medical University, No 81 Meishan Road, 230032 Hefei, Anhui China

**Keywords:** Stressful life events, Perinatal comorbid anxiety and depression, Adverse childhood experiences, Joint effects, Longitudinal study

## Abstract

**Background:**

Stressful life events (SLEs) and adverse childhood experiences (ACEs) have been reported to be associated with perinatal depression (PND) or perinatal anxiety (PNA) alone; however, in most cases, majority of PND and PNA coexist and could lead to more serious health consequences. The independent effect of recent SLEs and their joint effects with ACEs on perinatal comorbid anxiety and depression (CAD) remain inadequately explored.

**Methods:**

Based on a longitudinal study, 1082 participants receiving prenatal care in Ma’anshan, China were included. Women were recruited in the first trimester (T1: ≤14^+ 6^ weeks) and followed up at 15 ~ 27 weeks (T2), 28 ~ 40 weeks (T3), and postpartum (T4). Depression and anxiety status were assessed at all time points, while recent SLEs and ACEs were measured at T1. Logistic regression was conducted to examine the associations of SLEs with the risks of CAD at different time points, as well as their joint effects with ACEs on CAD.

**Results:**

Approximately 38.5% of women experienced at least one SLE, which was significantly associated with higher risks of CAD at all time points (*p* < 0.05). As the number of SLEs increased, the risk of CAD increased (*p* for trend < 0.05). Specific types of SLEs were associated with CAD in different periods, while only interpersonal events were consistently associated with risks of CAD throughout the whole perinatal period. The joint effects of SLEs with ACEs on CAD were identified throughout the perinatal period, with the highest observed in the first trimester (a*OR* = 7.47, 95% *CI*: 3.73–14.95; *p* for trend < 0.001).

**Conclusion:**

Our study demonstrated independent associations of recent SLEs and their joint effects with ACEs with risks of perinatal CAD. SLEs combined with ACEs should be recognized as a major risk factor for perinatal CAD and managed at the earliest time to prevent and control CAD.

**Supplementary Information:**

The online version contains supplementary material available at 10.1186/s12884-023-05375-1.

## Background

Stressful life events (SLEs), which are also called stressors, refer to harmful or threatening events occurring in one’s life, such as unemployment, the death of a loved one, or being diagnosed with severe disease [1]. SLEs are common, with 30–40% of the general population reporting at least one major SLE in the past year [2, 3], and they have been reported to be associated with increased risks of multiple health conditions, such as mental illness, coronary heart disease, and infectious diseases, etc. [1].

Accumulating evidence indicates that recent SLEs play an important role in the onset of perinatal depression (PND) and perinatal anxiety (PNA) [4–8], which are the most common complications during the perinatal period and could lead to both short- and long-term harmful health consequences for women themselves and their offspring [9–11]. However, earlier studies mainly examined the association of SLE with either PND or PNA alone. To our knowledge, no study to date has investigated the effects of recent SLEs on perinatal comorbid anxiety and depression (CAD). However, symptoms of perinatal anxiety and depression usually coexist [12, 13] and CAD has a high prevalence, ranging from 5%~26.9% during pregnancy and 2%~13% during the postpartum period [12, 14]. More significantly, CAD may lead to a higher risk of preterm birth, low birth weight, and small for gestational age than either PND or PNA alone [15, 16]. To this end, the association of recent SLEs with perinatal CAD warrants more attention from the research community.

In addition to recent SLEs, adverse childhood experiences (ACEs), which are generally conceptualized as stressful early life events, have been closely linked to PND or PNA [17, 18]. ACEs are defined as traumatic experiences that occur before 18 years including exposures to abuse, neglect, and household dysfunction [19], with 40–60% of pregnant women reporting at least one ACE [20–23]. A few studies have paid attention to the modifying effects of ACEs on SLEs regarding antenatal depression, and have suggested that ACEs could make pregnant women who have experienced SLEs more vulnerable to antenatal depression [24, 25]. However, no study has considered the two factors together in relation to CAD.

Based on the multihit hypothesis, also known as the cumulative stress hypothesis, which proposes that neuropsychiatric disorders may be triggered by a combination of two or more major adverse events [26], and that the prevalence of depression and anxiety was higher in individuals who reported exposure to both SLEs and ACEs [27, 28], we hypothesized that subjects with early adversity were more likely to experience perinatal CAD symptoms when they were previously exposed to one or more SLEs. The findings of studies conducted in the general adult population also support our hypothesis [27, 28]. However, we are not aware of any study that has investigated the joint effects of ACEs with SLEs on perinatal CAD, which could contribute to identifying higher risk populations. Thus, we used data from a longitudinal study with up to four repeated CAD measurements from the first trimester of pregnancy to postpartum. We aimed to (1) clarify the associations between recent SLEs and CAD at different time points throughout the perinatal period, and (2) examine the joint effects of ACEs with recent SLEs on CAD.

## Methods

### Study design and settings

This study draws on data from a pilot study of an implementation research [29] which was conducted at Ma’anshan Maternal and Child Health Center, of the Perinatal Depression Screening and Management program (PDSM). The PDSM aims to establish an effective perinatal depression screening and management system within primary health care system settings in China. Our current study adopted a longitudinal cohort design. At Ma’anshan Maternal and Child Health Center, registered pregnant women were continuously recruited at their first prenatal visit (T1: in the first trimester, ≤ 14^+ 6^ weeks), and were followed up at 15 ~ 27 weeks (T2) and 28 ~ 40 weeks (T3) of pregnancy and within 1 year postpartum (T4). Depression and anxiety were assessed at all time points, while recent SLEs and ACEs were measured at T1.

### Participants

**Fig. 1:**
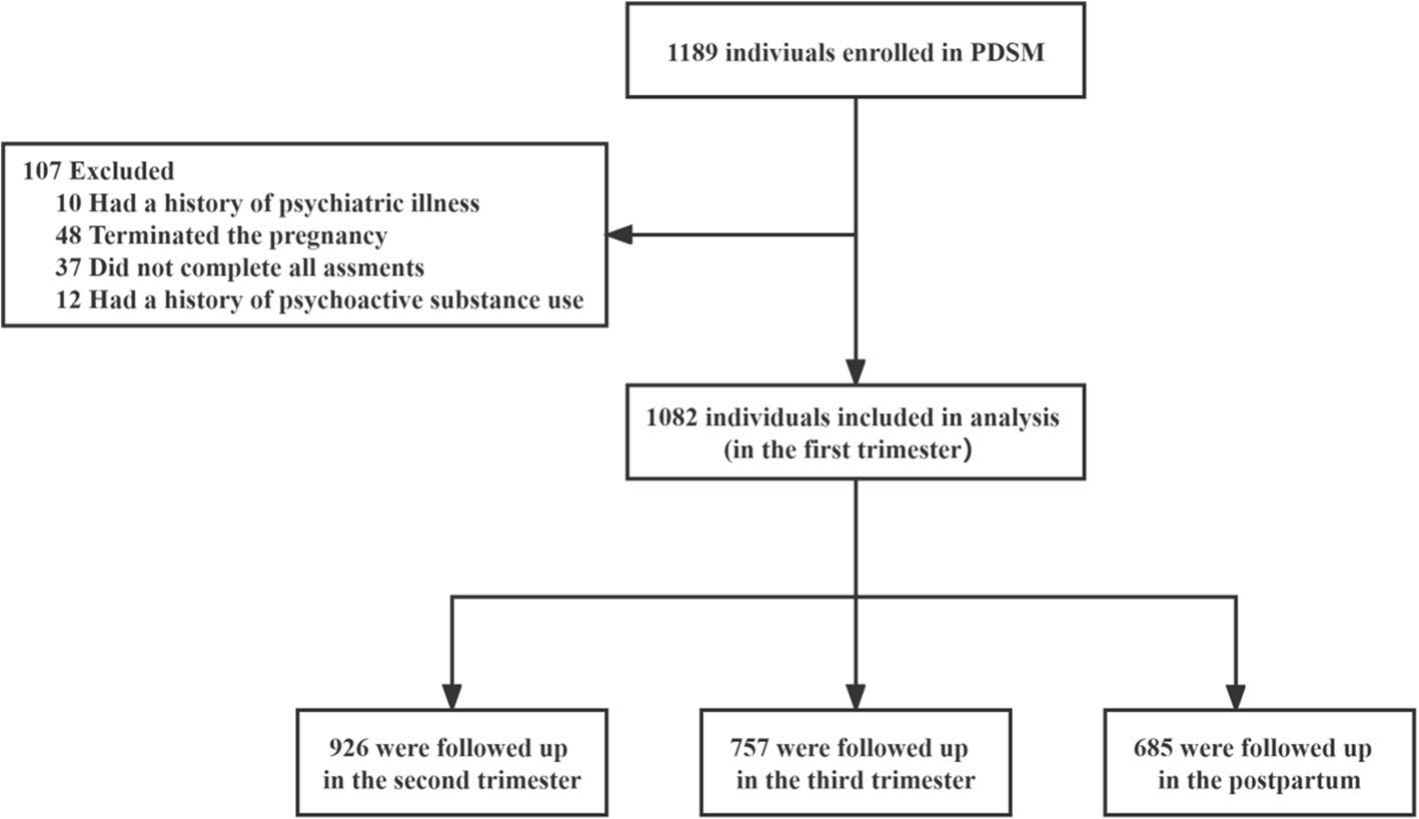
Consort diagram of participants included in analytic data set. PDSM, Perinatal Depression Screening and Management program

From May 2019 to December 2019, pregnant women were recruited when they received their first prenatal care. The inclusion criteria were as follows: (1) ≤ 14^+ 6^ weeks pregnant; (2) 18 years or older; and (3) able to complete questionnaires independently. Considering that a history of psychoactive substances, psychiatric illness, and termination of pregnancy can be deemed as “severe life events” for specific pregnant women, which is one of the most consistent predictors of depression or anxiety, to avoid overestimating effects, we excluded participants with a history of psychoactive substance, a history of psychiatric illness, and termination of pregnancy and those who did not complete all assessments (such as missing data about SLEs). Finally, 1082 participants in the first trimester were included in this second analysis. Among the 1082 individuals, 926, 757, and 685 had depression and anxiety data at T2, T3, and T4, respectively. The flow chart of the exclusion process is presented in Fig. 1. This study obtained ethics approval from Anhui Medical University Biomedical Ethics Committee [20170358]. This research was carried out in accordance with the Declaration of Helsinki. Written informed consent was obtained from all participants.

### Measurements

#### Recent stressful life events and adverse childhood experiences

Recent SLEs were collected when recruited using a 19-item self-report questionnaire adapted from the Life Events Scale for Pregnant Women (LESPW) compiled by Yan Gao et al. [30]. However, life events in the LESPW were not classified; therefore, according to other studies [8, 31–34], we grouped the 19 items into five categories: (1) interpersonal life events (e.g., separation from husband, bad relationship with family/neighbor); (2) changes in the health of self or partner (e.g., serious illness of self, injuries to husband); (3) family (except for the husband) related events (e.g., serious illness, death or gambling of a loved one); (4) financial crisis (e.g., loss of a job, investment failure, property loss due to theft or a scam); or (5) residential relocation or unexpected scare. Participants were asked if they had experienced any of these life events in the past year. Responses to each item were presented in a “yes” or “no” format (no = 0, yes = 1). If the participants had responded yes to one or more items within one category, the code = 1 was assigned to this category; otherwise, it was 0.

ACEs were measured by a 10-item self-report questionnaire that was applied in the CDC-Kaiser ACE Study in America [35] and has also been used in the Chinese population with validated reliability [36, 37]. Participants were asked if they had experienced one or more childhood events. Responses to each item were coded as “yes” or “no” to indicate the presence or absence of the experience (no = 0, yes = 1).

### Comorbid anxiety and depression

Symptoms of depression were rated by the Edinburgh Postnatal Depression Scale (EPDS), which contains 10 items. All items are scored from 0 to 3, thus producing a maximum score of 30. The EPDS has been extensively used as a measuring tool for perinatal depression with good internal validity (Cronbach’s alpha is 0.82) [38, 39]. A standard cut-off score ≥ 9 was used to indicate perinatal depression [40].

The Generalized Anxiety Disorder Scale 7 Item (GAD-7) was used to measure anxiety symptoms [41]. The GAD-7 has been validated in both pregnancy and the postpartum period [42], and it has been shown to have good internal consistency reliability and validity among pregnant Chinese women (Cronbach’s alpha is 0.84) [43]. Zero to 3 was assigned to each item and the overall score ranged from 0 to 21. The higher the score, the more severe the anxiety symptoms. To indicate probable anxiety, a cutoff score ≥ 5 was used [44, 45].

Therefore, women were considered to have CAD symptoms if their EPDS scores were no less than 9 scores and their GAD-7 scores were no less than 5 scores.

### Covariates

A self-structured questionnaire was used to gather information on sociodemographic characteristics (e.g., maternal age, residence, marital status, education), lifestyle habits (e.g., smoking, passive smoking, alcohol use), and health-related factors (e.g., reproductive intention, conception, parity). Smoking was defined as having smoked at least 100 cigarettes a year, and if the person was passively exposed to smoking at least once a week in the past year, it was regarded as passive smoking. Alcohol use referred to drinking at least 1 ~ 3 times a month (one alcohol use was defined as 340 ml of beer, 140 ml of wine or 43 ml of liquor).

### Statistical analysis

The mean and standard deviations were used to describe continuous variables, and the categorical data were presented as frequencies and percentages. The baseline characteristics of the participants among the different groups were compared using the chi-square test for categorical variables and ANOVA for continuous variables. Multivariate logistic regression was conducted to estimate whether if recent SLEs (including different numbers and categories) were associated with CAD, and whether there were joint effects of SLEs with ACEs on the risk of CAD throughout the whole perinatal period. A linear mixed model was performed to analyze the association between exposure to SLEs and ACEs with the sum score of EPDS and GAD-7 across the entire perinatal period. Based on the chi-square tests and ANOVA, adjusted covariates with *p* values less than 0.05 were selected. Of note, considering that some participants received intervention from the PDSM program, this factor was also incorporated as a covariate. Odds ratios (ORs) and corresponding 95% confidence intervals (CIs) were calculated to estimate the magnitude of the associations. To estimate the impact of missing data during follow-up, we performed a sensitivity analysis after multiple imputation. All analyses were conducted by using IBM SPSS Statistics version 22.0 and we used GraphPad Prism version 6.0 to draw forest plots of the ORs and 95% CIs.

## Results

### Sociodemographic characteristics of the participants

The sociodemographic data of the participants are summarized in Table 1. The mean age of 1082 participants was 28.69 (4.01). Most of the pregnant women were living in urban areas (87.5%) and were married (93.8%). The participants were highly educated, with 63.8% having a junior college/regular college degree or above. There were differences in maternal age, marital status, parity and unexpected pregnancy between the CAD and neither depression nor anxiety group.

**Table 1:** Demographic characteristics and life styles of women in perinatal period according to different depression and anxiety status

Characteristics	Total	No depression and anxiety (*n* = 561)	Only depression (*n* = 41)	Only anxiety (*n* = 228)	CAD (*n* = 252)	*χ*^2^/*F*^a^	*p*
Age (years), mean (SD)	28.69 (4.01)	28.95 (4.01)	28.14 (3.97)	28.76 (3.85)	28.13 (4.10)	2.73	**0.043**
Residence						3.46	0.326
Urban	947 (87.5)	499 (88.9)	33 (80.5)	198 (86.8)	217(86.1)		
Rural	135 (12.5)	62 (11.1)	8 (19.5)	30 (13.2)	35 (13.9)		
Marital status						16.20	**0.001**
Married	1015 (93.8)	534 (95.2)	39 (95.1)	219 (96.1)	223 (88.5)		
Unmarried or others	67 (6.2)	27 (4.8)	2 (4.9)	9 (3.9)	29 (11.5)		
Education status						12.57	**0.050**
Middle school or below	181 (16.7)	100 (17.8)	10 (24.4)	22 (9.7)	49 (19.4)		
High school or technical secondary school	211 (19.5)	109 (19.5)	9 (22.0)	45 (19.7)	48 (19.1)		
Junior college /regular college or above	690 (63.8)	352 (62.7)	22 (53.6)	161 (70.6)	155 (61.5)		
Annual household income (¥)						7.32	0.292
< 50 K	114 (10.5)	55 (9.8)	6 (14.6)	17 (7.5)	36 (14.3)		
50 K ~ 200 K	815 (75.3)	424 (75.6)	29 (70.7)	179 (78.5)	183 (72.6)		
> 200 K	153(14.2)	82 (14.6)	6 (14.7)	32 (14.0)	33 (13.1)		
Work status						19.73	**0.020**
Unemployed or resign	446 (41.2)	229 (40.8)	21 (51.2)	77 (33.8)	119 (47.2)		
Paid leave	60 (5.5)	26 (4.6)	4 (9.8)	12 (5.3)	18 (7.1)		
Part-time job	23 (2.1)	13 (2.4)	0 (0.0)	3 (1.3)	7 (2.8)		
Full-time job	553 (51.1)	293 (52.2)	16 (39.0)	136 (59.6)	108 (42.9)		
Unexpected pregnancy						10.95	**0.012**
Yes	231 (21.3)	107 (19.1)	10 (24.4)	42 (18.4)	72 (28.6)		
No	851 (78.7)	454 (80.9)	31 (75.6)	186 (81.6)	180 (71.4)		
Conception						1.02	0.796
Natural	1009 (93.3)	522 (93.0)	37 (90.2)	215 (94.3)	235 (93.3)		
Assisted	73 (6.7)	39 (7.0)	4 (9.8)	13 (5.7)	17 (6.7)		
Parity						9.02	**0.028**
0	696 (64.3)	341 (60.8)	28 (68.3)	164 (71.9)	163 (64.7)		
≥ 1	386 (35.7)	220 (39.2)	13 (31.7)	64 (28.1)	89 (35.3)		
Smoking						2.20	0.532
Yes	46 (4.3)	23 (4.1)	0 (0.0)	11 (4.8)	12 (4.8)		
No	1036 (95.7)	538 (95.9)	41 (100.0)	217 (95.2)	240 (95.2)		
Passive smoking in the past year						7.06	0.070
Yes	384 (35.5)	183 (32.6)	11 (26.8)	88 (38.6)	102 (40.5)		
No	698 (64.5)	378 (67.4)	30 (73.2)	140 (61.4)	150 (59.5)		
Alcohol use						10.66	**0.014**
Yes	144 (13.3)	65 (11.6)	10 (24.4)	25 (11.0)	44 (17.5)		
No	938 (86.7)	496 (88.4)	31 (75.6)	203 (89.0)	208 (82.5)		
Pre-pregnancy BMI (kg/m^2^)						8.77	0.187
< 18.5	169 (15.6)	77 (13.7)	7 (4.1)	35 (15.4)	50 (19.9)		
18.5 ~ 23.9	685 (63.3)	355 (63.3)	29 (70.7)	151 (66.2)	150 (59.5)		
≥ 24.0	228 (21.1)	129 (23.0)	5 (12.2)	42 (18.4)	52 (20.6)		

Data are presented as n (%) or the mean (standard deviation)

*SD* standard deviation, *CAD* co-morbid anxiety and depression, *BMI* body mass index

^a^*χ*^*2*^ = chi-square test; *F* = ANOVA

### Relationship between recent SLEs and perinatal CAD

As shown in Tables 2 and 38.5% (417/1082) of pregnant women reported at least one SLE in the past year. The prevalence of symptoms of CAD was 14.9% in the first trimester, 5.4% in the second trimester, 4.6% in the third trimester, and 7.1% in the postpartum period.

**Table 2:** Associations between recent SLEs and perinatal CAD

Period	Group	Total N (%)	SLE		Model 1^a,c^		Model 2^b,c^	
			YES	NO	*OR* (95%*CI*)	*p*	a*OR* (95%CI)	*p*
First trimester	No depression and anxiety	661 (61.1)	215 (51.6)	446 (67.1)	Reference		Reference	
	Only depression	31 (2.9)	12 (2.9)	19 (2.9)	1.31 (0.63–2.75)	0.475	1.23 (0.54–2.81)	0.626
	Only anxiety	229 (21.1)	98 (23.5)	131 (19.7)	1.55 (1.14–2.11)	0.005	1.73 (1.24–2.42)	0.001
	Co-morbidity	161 (14.9)	92 (22.0)	69 (6.3)	2.77 (1.95–3.93)	< 0.001	2.36 (1.47–3.81)	< 0.001
Second trimester	No depression and anxiety	768 (82.9)	267 (76.7)	501 (86.7)	Reference		Reference	
	Only depression	11 (1.2)	6 (1.7)	5 (0.9)	2.25 (0.68–7.45)	0.183	1.52 (0.41–5.61)	0.529
	Only anxiety	97 (10.5)	45 (13.0)	52 (9.0)	1.62 (1.06–2.49)	0.026	1.84 (1.16–2.90)	0.009
	Co-morbidity	50 (5.4)	30 (8.6)	20 (3.4)	2.82 (1.57–5.05)	0.001	2.33 (1.21–4.48)	0.011
Third trimester	No depression and anxiety	625 (82.6)	226 (76.4)	399 (86.6)	Reference		Reference	
	Only depression	15 (2.0)	9 (3.0)	6 (1.3)	2.65 (0.93–7.54)	0.068	2.03 (0.65–6.29)	0.221
	Only anxiety	82 (10.8)	41 (13.9)	41 (8.9)	1.77 (1.11–2.80)	0.016	2.12 (1.28–3.52)	0.004
	Co-morbidity	35 (4.6)	20 (6.7)	15 (3.2)	2.36 (1.18–4.69)	0.015	2.81 (1.32–6.01)	0.008
Postpartum	No depression and anxiety	568 (82.9)	202 (76.5)	366 (86.9)	Reference		Reference	
	Only depression	10 (1.5)	3 (1.1)	7 (1.7)	0.78 (0.20–3.04)	0.716	0.45 (0.10–1.95)	0.284
	Only anxiety	58 (8.5)	29 (11.0)	29 (6.9)	1.81 (1.05–3.12)	0.032	1.97 (1.10–3.56)	0.024
	Co-morbidity	49 (7.1)	30 (11.4)	19 (4.5)	2.86 (1.57–5.21)	0.001	3.23 (1.67–6.23)	< 0.001

Model 1 was unadjusted

Model 2 was adjusted for age, marital status, education status, work status, parity, unexpected pregnancy, alcohol use and intervention

*SLEs* stressful life events, *CAD* co-morbid anxiety and depression, *OR* odds ratio, *CI* confidence interval

Based on logistic regression analysis, after adjusting for sociodemographic, intervention, lifestyle, and health-related factors (Model 2), there were significant associations of recent SLEs with CAD (*p* < 0.05) from the first trimester to postpartum, and the largest magnitude of association was observed at postpartum, with the probability of CAD increasing up to 3.23 times. The details are shown in Table 2.

### Associations of the number of recent SLEs with CAD

As shown in Table 3, among the 1082 participants, 271 (25.0%) reported one SLE, 87 (8.0%) experienced two SLEs and 59 (5.5%) reported three or more SLEs. A dose-response association was observed between recent SLEs and CAD throughout the whole perinatal period (*p* for trend < 0.05); as the number of SLEs increased, the probability of CAD increased. Compared with individuals who experienced no SLEs, those who experienced three or more events had the highest probability of CAD, with the largest magnitude of association observed in the first trimester (a*OR* = 11.75; 95% *CI*: 4.94–27.95).

**Table 3:** Associations between the number of recent SLEs and perinatal CAD

Number of SLEs	Total *N* = 1082 (%)	CAD in the first trimester^a^	CAD in the second trimester^a^	CAD in the third trimester^a^	CAD in the postpartum^a^
		a*OR (*95%*CI)*	a*OR* (95%*CI)*	a*OR* (95%*CI)*	a*OR* (95%*CI)*
0	665 (61.5)	Reference	Reference	Reference	Reference
1	271 (25.0)	1.66 (0.95–2.90)	1.75 (0.80–3.86)	2.32 (0.99–5.47)	2.29 (1.07–4.91)^*^
2	87 (8.0)	2.03 (0.89–4.63)	3.12 (1.22–7.95)^*^	3.48 (1.11–10.90)^*^	5.40 (2.17–13.47)^***^
≥ 3	59 (5.5)	11.75 (4.94–27.95)^***^	3.33 (1.19–9.34)^*^	4.96 (1.32–18.62)^**^	5.89 (1.87–18.55)^**^
*p* for trend	-	< 0.001	0.003	0.003	< 0.001

*SLEs* stressful life events, *CAD* co-morbid anxiety and depression, *OR* odds ratio, *CI* confidence interval

^*^p < 0.05, ^**^p < 0.01, ^***^p < 0.001, a*OR* was adjusted for age, marital status, education status, work status, parity, unexpected pregnancy, alcohol use and intervention

^a^Neither depression nor anxiety as a reference

### Associations of the types of recent SLEs with perinatal CAD

**Fig. 2:**
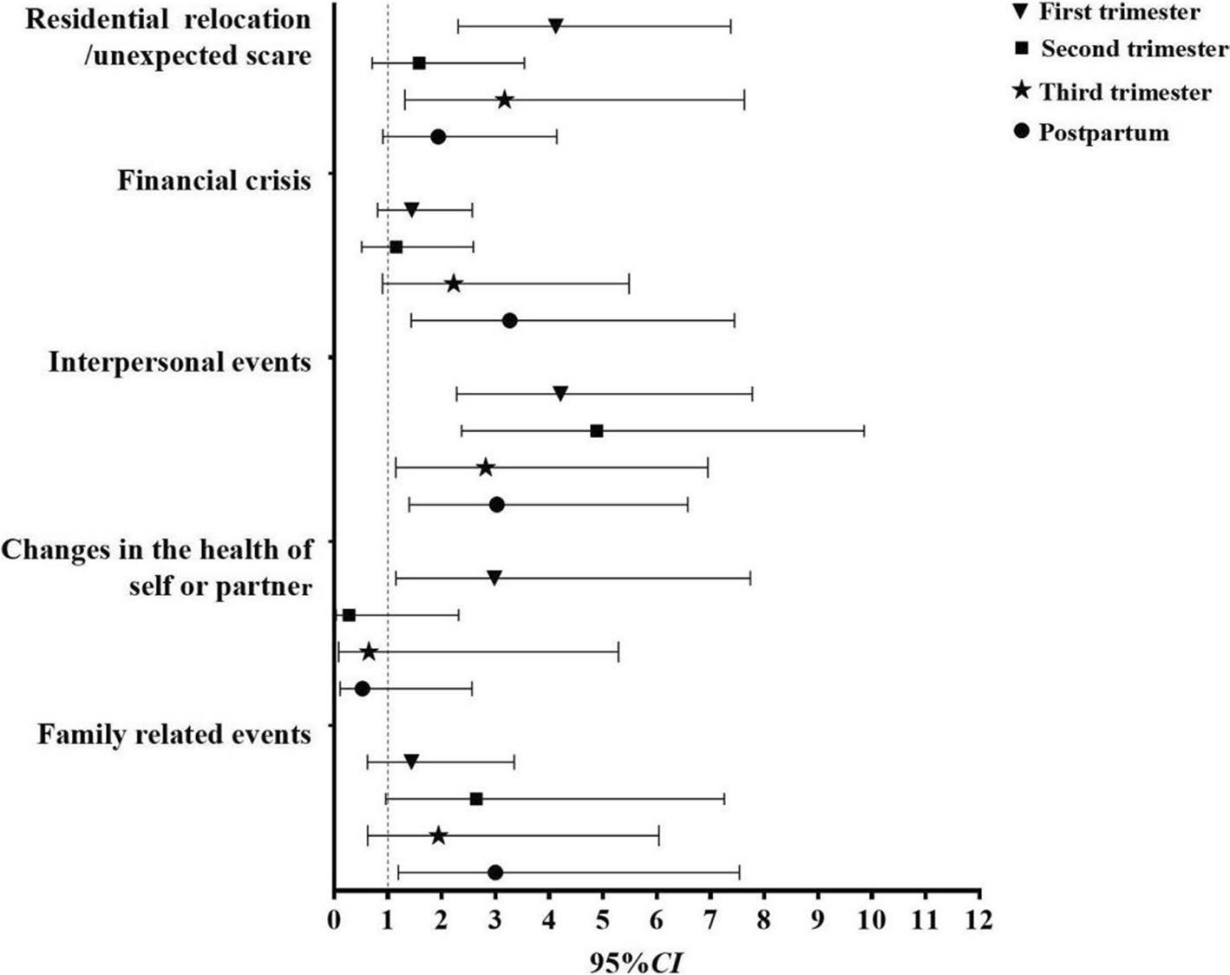
Associations of five categories of recent SLEs with perinatal CAD. Model was adjusted for age, marital status, education status, work status, parity, unexpected pregnancy, alcohol use, and intervention

Figure 2 shows the associations of the five categories of SLEs with CAD from the first trimester to postpartum. Different categories of SLEs were associated with CAD in specific periods. Family-related events and financial crises were only associated with an increased probability of CAD in the postpartum period (a*OR =* 3.00, 95% *CI*: 1.19–7.54; a*OR =* 3.27; 95% *CI*: 1.43–7.44); changes in the health of self or partner were only linked to CAD in the first trimester (a*OR =* 2.98, 95% *CI*: 1.15–7.74); and residential relocation/unexpected scare was associated with CAD in the first and third trimesters (a*OR* = 4.12, 95% *CI*: 2.30–7.37; a*OR* = 3.17, 95% *CI*: 1.32–7.63). Interpersonal SLEs were consistently associated with increased probabilities of CAD throughout the whole perinatal period (*p <* 0.05).

### Joint effects of recent SLEs and ACEs on perinatal CAD

Table 4 shows the results of joint effects of SLEs and ACEs on CAD. As shown, 10.5% (114/1082) of the participants with ACEs also experienced SLEs. Compared with women experiencing neither recent SLEs nor ACEs, those who experienced either SLEs or ACEs alone had an increased probability of CAD; and women who experienced both ACEs and recent SLEs had the highest probability for CAD at all four-time points, with an increased probability ranging from 3.12 to 7.47 times (*p* < 0.05). The mean EPDS plus GAD-7 scores of women with ACEs and SLEs were 10.95 (6.96) and 6.22 (4.97) for those without either ACEs or SLEs, with a significant group difference being observed (LS means = 3.51, 95% *CI*: 2.63–4.38; *p <* 0.001 ) (shown in Table S1).

**Table 4:** Joint effects of ACEs with recent SLEs on perinatal CAD

Group	Total N = 1082 (%)	CAD in the first trimester	CAD in the second trimester	CAD in the third trimester	CAD in the postpartum
		a*OR* (95%*CI)*	a*OR* (95%*CI)*	a*OR* (95%*CI)*	a*OR* (95%*CI)*
No SLE and ACE	576 (53.2)	Reference	Reference	Reference	Reference
Only SLE	303 (28.0)	1.89 (1.07–3.33)^**^	2.50 (1.16–5.39)^**^	2.30 (0.98–5.37)	2.37 (1.10–5.11)^*^
Only ACE	89 (8.2)	2.80 (1.27–6.20)^*^	2.01 (0.68–5.95)	0.72 (0.15–3.52)	1.30 (0.35–4.80)
SLE and ACE	114 (10.5)	7.47 (3.73–14.95)^***^	3.12 (1.19–8.20)^**^	4.09 (1.34–12.43)^*^	7.36 (3.04–17.79)^***^
*p* for trend	-	< 0.001	0.005	0.008	< 0.001

*ACEs* adverse childhood experiences, *SLEs* stressful life events, *CAD* co-morbid anxiety and depression, *OR* odds ratio, *CI* confidence interval

^*^p < 0.05, ^**^p < 0.01, ^***^p < 0.001, a*OR* was adjusted for age, marital status, education status, work status, parity, unexpected pregnancy, alcohol use and intervention

### Sensitivity analysis

The sensitivity analyses showed the stability of our results (Supplemental Tables S2-S4).

## Discussion

Our current longitudinal study suggested that recent SLEs were significantly associated with CAD at all time points throughout the whole perinatal period in a dose-response manner. More importantly, the joint effects of ACEs with recent SLEs on perinatal CAD were observed. The results of our study highlight the vulnerability to CAD among women who had ACEs and experienced recent SLEs.

Consistent with the findings of previous research [46], our study also found that the risk of CAD increased with the cumulative numbers of SLEs, with pregnant women reporting three or more SLEs being nearly five to eleven times more likely to develop CAD. Our findings along with others support the idea that the impacts of SLEs on psychopathology appear to be dose-dependent [47]. Although the underlying mechanisms of recent SLEs on perinatal CAD are unclear, the findings of studies examining the potential mechanism of stress or SLEs on mental health (e.g. depression or anxiety) may provide insight into this. For example, a possible explanation might be that altered maternal stress levels as a result of SLEs exposure could disturb individual homeostasis through inflammation, activation of the HPA axis, and dysregulation of intestinal microbiota, which may potentially contribute to the triggering of perinatal mental illness [48, 49]. In addition, it is noteworthy that SLEs could also interact with the genetic background to influence the risk of mental health issues; for example, a study indicated that individuals who carry certain alleles (e.g.,the serotonin-transporter-linked polymorphic region, 5-HTTLPR) might be pose susceptible to the development of psychopathology when faced with environmental adversity (e.g., SLEs) [50].

Significantly, not all domains of SLEs contributed equally to CAD in the perinatal period. Consistent with previous studies that interpersonal stressful life events may be a stronger predictor of depression onset than other types of life events [51], our study also found that the association of interpersonal SLEs with perinatal CAD was the most stable,and among 38.5% of pregnant women who experienced at least one SLE in the past year, one-third had interpersonal SLEs. Fortunately, despite some real-life stressors occurring out of an individual’s control, such as the death of a loved one, an unexpected scare, or a job loss, the adverse impacts of SLEs can be ameliorated by implementing an intervention. The “stress-buffering hypothesis” proposes that supportive interactions can act as a buffer against the negative consequences of SLEs on health [52], such as social support [46, 53]. Therefore, more studies are needed to specify the role of social support among women exposed to ACEs and SLEs in preventing or reducing CAD symptoms.

Our findings support the “multihit hypothesis”. In concordance with the finding of Evans MG et al. [25], individuals with ACEs who also experienced recent SLEs were at a greater risk of perinatal CAD, which also aligns with the previous findings that the accumulation of adversity in childhood and adulthood is more damaging [24]. One possible explanation for this is that exposure to ACEs increases vulnerability to the effects of later SLEs, which is called stress sensitization effects [3]. Specifically, childhood adversities can “sensitize” individuals to psychopathology by lowering their tolerance to relatively minor stressors [3]. Thus, our study supports adopting screening for recent SLEs and ACEs in order to prevent and control perinatal CAD, which may help identify the most vulnerable women.

To the best of our knowledge, this is the first study to examine the joint effects of recent SLEs with ACEs on CAD throughout the whole perinatal period, with up to four repeated measurements of CAD. However, some limitations should be acknowledged. First,, data on SLEs and ACEs were collected retrospectively; thus, recall bias could not be fully avoided. Second, we excluded participants with a history of psychoactive substance use and psychiatric illness, and those who terminated their pregnancy, and thus excluded many patients who may have at higher risk of CAD symptoms, which could underestimate the relationship among SLEs, ACEs, and CAD. Third, although the EPDS and GAD-7 are well validated and widely used in perinatal women, they are not the only screening tool for depression or anxiety symptoms. Finally, the willingness to report stressful experiences among currently (at the time of the interview) depressed or anxious versus nondepressed and relaxed respondents can be considerable [53], which may bias the association.

## Conclusion

Our longitudinal study suggested recent SLEs were associated with increased risks of CAD throughout the whole perinatal period, with joint effects with ACEs being observed. Thus, we recommend health care workers engage in routine screening for SLEs and ACEs early in pregnancy to identify those who are at the highest risk for depression and anxiety and deliver targeted interventions to prevent and manage CAD in a timely fashion. In the future, more studies are needed to specify the role of social support among women exposed to ACEs and SLEs in order to prevent or reduce CAD symptoms.

## Supplementary Information

**Additional file 1.**

## Data Availability

The datasets generated during the current study are not publicly available due to the findings of the pilot study have not been published, but are available from the corresponding author on reasonable request.

## Abbreviations

SLE: Stressful life events
ACE: adverse childhood experiences
PND: perinatal depression
PNA: perinatal anxiety
CAD: co-morbid anxiety and depression
PDSM: Perinatal Depression Screening and Management program
LESPW: Life Events Scale for Pregnant Women
EPDS: Edinburgh Postnatal Depression Scale
GAD-7: Generalized Anxiety Disorder Scale 7 Item
OR: Odds ratios
CI: confidence intervals
5-HTTLPR: serotonin-transporter-linked polymorphic region
