# Supplementary material for: Joint effects of recent stressful life events and adverse childhood experiences on perinatal comorbid anxiety and depression
Source: BMC Pregnancy Childbirth. 2023 Jan 18;23:41. doi: 10.1186/s12884-023-05375-1 (PMC9847044; doi:10.1186/s12884-023-05375-1)
Supplement: Supplementary file 1 — Additional file 1. [file 12884_2023_5375_MOESM1_ESM.docx]

**Supplementary materials for results**

**Joint effects of recent stressful life events with adverse childhood experiences on perinatal co-morbid anxiety and depression**

Yanyan Hou, Mengqing Shang, Xiayan Yu , Yue Gu , Haiyan Li, Mengjuan Lu, Minmin Jiang , Hualong Zhen, Beibei Zhu^*^ and Fangbiao Tao ^*^

| **Table S1 Differences in the sum score of EPDS plus GAD-7 between women with SLEs and ACEs and women without either SLEs or ACEs** | | | | | | | | |
| --- | --- | --- | --- | --- | --- | --- | --- | --- |
| **Group** | **Mean score of EPDS plus GAD-7** | **Model 1** | | |  | **Model 2** | | |
|  |  | **LS means** | **95% *CI*** | ***p*** |  | **LS means** | **95% *CI*** | ***p*** |
| No SLE and ACE | 6.22 (4.97) | reference |  |  |  | reference |  |  |
| Only SLE | 8.02 (5.88) | 1.75 | 1.15-2.35 | <0.001 |  | 1.62 | 1.02-2.22 | <0.001 |
| Only ACE | 8.07 (5.74) | 1.61 | 0.66-2.57 | 0.001 |  | 1.12 | 0.19-2.04 | 0.008 |
| SLE and ACE | 10.95 (6.96) | 4.10 | 3.22-4.97 | <0.001 |  | 3.51 | 2.63-4.38 | <0.001 |
| Model 1 was unadjusted  Model 2 was adjusted for age, marital status, education status, work status, parity, unexpected pregnancy, alcohol use and intervention. | | | | | | | | |

**Sensitive analysis**

**Analysis were performed after multiple imputation of missing data on EPDS scores and GAD-7 scores during follow-up.**

| **Table S2 Associations between recent SLEs and perinatal CAD after multiple imputation** | | | | | | | | | | |
| --- | --- | --- | --- | --- | --- | --- | --- | --- | --- | --- |
| **Period** | **Group** | **Total**  **N (%)** | **SLE** | |  | **Model 1^a,c^** | |  | **Model 2^b,c^** | |
|  |  |  | **YES** | **NO** |  | **OR (95%CI)** | ***p*** |  | **aO*R* (95%CI)** | ***p*** |
| First trimester | No depression and anxiety | 661 (61.1) | 215 (51.6) | 446 (67.1) |  | Reference |  |  | Reference |  |
|  | Only depression | 31 (2.9) | 12 (2.9) | 19 (2.9) |  | 1.31 (0.63-2.75) | 0.475 |  | 1.23 (0.54-2.81) | 0.626 |
|  | Only anxiety | 229 (21.1) | 98 (23.5) | 131 (19.7) |  | 1.55 (1.14-2.11) | 0.005 |  | 1.73 (1.24-2.42) | 0.001 |
|  | Co-morbidity | 161 (14.9) | 92 (22.0) | 69 (6.3) |  | 2.77 (1.95-3.93) | <0.001 |  | 2.36 (1.47-3.81) | <0.001 |
| Second trimester | No depression and anxiety | 885 (81.8) | 318 (76.3) | 567 (85.3) |  | Reference |  |  | Reference |  |
|  | Only depression | 19 (1.8) | 10 (2.4) | 9 (1.4) |  | 1.98 (0.80-4.93) | 0.141 |  | 1.28 (0.47-3.46) | 0.632 |
|  | Only anxiety | 127 (11.7) | 58 (13.9) | 69 (10.4) |  | 1.50 (1.03-2.18) | 0.035 |  | 1.55 (1.04-2.32) | 0.032 |
|  | Co-morbidity | 51 (4.7) | 31 (7.4) | 20 (3.0) |  | 2.76 (1.55-4.93) | 0.001 |  | 2.34 (1.23-4.45) | 0.010 |
| Third trimester | No depression and anxiety | 853 (78.8) | 308 (73.9) | 545 (82.0) |  | Reference |  |  | Reference |  |
|  | Only depression | 45 (4.2) | 22 (5.3) | 23 (3.5) |  | 1.69 (0.93-3.09) | 0.086 |  | 1.43 (0.76-2.71) | 0.272 |
|  | Only anxiety | 140 (12.9) | 63 (15.1) | 77 (11.6) |  | 1.45 (1.01-2.08) | 0.045 |  | 1.49 (1.01-2.20) | 0.043 |
|  | Co-morbidity | 44 (4.1) | 24 (5.8) | 20 (3.0) |  | 2.12 (1.15-3.90) | 0.015 |  | 2.25 (1.17-4.34) | 0.015 |
| Postpartum | No depression and anxiety | 803 (74.2) | 288 (69.1) | 515 (77.4) |  | Reference |  |  | Reference |  |
|  | Only depression | 54 (5.0) | 19 (4.6) | 35 (5.3) |  | 0.97 (0.55-1.73) | 0.920 |  | 0.71 (0.39-1.31) | 0.276 |
|  | Only anxiety | 145 (13.4) | 65 (15.6) | 80 (12.0) |  | 1.45 (1.02-2.08) | 0.041 |  | 1.51 (1.03-2.22) | 0.035 |
|  | Co-morbidity | 80 (7.4) | 45 (10.8) | 35 (5.3) |  | 2.30 (1.45-3.66) | 0.015 |  | 2.51 (1.51-4.17) | <0.001 |
| SLEs stressful life events, CAD co-morbid anxiety and depression, OR odds ratio, CI confidence interval  Model 1 was unadjusted  Model 2 was adjusted for age, marital status, education status, work status, parity, unexpected pregnancy, alcohol use and intervention | | | | | | | | | | |

| **Table S3 Associations between the number of recent SLEs and perinatal CAD after multiple imputation** | | | | | | | |
| --- | --- | --- | --- | --- | --- | --- | --- |
| **Number of SLEs** | **CAD in the first trimester^a^** |  | **CAD in the second trimester^a^** |  | **CAD in the third trimester^a^** |  | **CAD in the postpartum^a^** |
|  | ***OR (*95%*CI)*** |  | ***aOR* (95%*CI)*** |  | ***aOR* (95%*CI）*** |  | ***aOR* (95%*CI)*** |
| 0 | Reference |  | Reference |  | Reference |  | Reference |
| 1 | 1.66 (0.95-2.90) |  | 1.80 (0.84-3.86) |  | 1.95 (0.92-4.14） |  | 1.82 (1.00-3.32) |
| 2 | 2.03 (0.89-4.63) |  | 3.23 (1.29-8.06)^*^ |  | 2.64 (0.97-7.18) |  | 5.19 (2.58-10.44)^***^ |
| ≥3 | 11.75 (4.94-27.95)^***^ |  | 3.20 (1.17-8.74)^*^ |  | 3.20 (1.05-9.75)^*^ |  | 2.42 (0.93-6.25) |
| *p* for trend | <0.001 |  | 0.003 |  | 0.010 |  | <0.001 |
| SLEs stressful life events, CAD co-morbid anxiety and depression, OR odds ratio, CI confidence interval  ^*^*p*<0.05, ^**^*p*<0.01, ^***^*p*<0.001; aOR was adjusted for age, marital status, education status, work status, parity, unexpected pregnancy, alcohol use and intervention  **^a^** Neither depression nor anxiety as a reference | | | | | | | |

| **Table S4 Joint effects of ACEs with recent SLEs on perinatal CAD after multiple imputation** | | | | | | | |
| --- | --- | --- | --- | --- | --- | --- | --- |
| **Group** | **CAD in the first trimester** |  | **CAD in the second trimester** |  | **CAD in the third trimester** |  | **CAD in the postpartum** |
|  | **a*OR* (95%*CI)*** |  | **a*OR* (95%*CI)*** |  | **a*OR* (95%*CI)*** |  | **a*OR* (95%*CI)*** |
| No SLE and ACE | Reference |  | Reference |  | Reference |  | Reference |
| Only SLE | 1.89 (1.07-3.33)^**^ |  | 2.25 (1.47-3.45)^***^ |  | 0.90 (0.25-3.27) |  | 1.55 (0.85-2.84) |
| Only ACE | 2.80 (1.27-6.20)^*^ |  | 1.94 (1.07-3.52)^*^ |  | 1.97 (0.93-4.18) |  | 0.63 (0.21-1.87) |
| SLE and ACE | 7.47 (3.73-14.95)^***^ |  | 2.61 (1.54-4.43)^***^ |  | 2.87 (1.14-7.22)^*^ |  | 5.12 (2.63-9.96)^***^ |
| *p* for trend | <0.001 |  | 0.006 |  | 0.014 |  | <0.001 |
| ACEs adverse childhood experiences, SLEs stressful life events, CAD co-morbid anxiety and depression, OR odds ratio, CI confidence interval  ^*^*p*<0.05, ^**^*p*<0.01, ^***^*p*<0.001, aOR was adjusted for age, marital status, education status, work status, parity, unexpected pregnancy, alcohol use and intervention | | | | | | | |
